# Imaging the passionate stage of romantic love by dopamine dynamics

**DOI:** 10.3389/fnhum.2015.00191

**Published:** 2015-04-09

**Authors:** Kayo Takahashi, Kei Mizuno, Akihiro T. Sasaki, Yasuhiro Wada, Masaaki Tanaka, Akira Ishii, Kanako Tajima, Naohiro Tsuyuguchi, Kyosuke Watanabe, Semir Zeki, Yasuyoshi Watanabe

**Affiliations:** ^1^Pathophysiological and Health Science Team, RIKEN Center for Life Science TechnologiesKobe, Japan; ^2^Department of Physiology, Osaka City University Graduate School of MedicineOsaka, Japan; ^3^Department of Medical Science on Fatigue, Osaka City University Graduate School of MedicineOsaka, Japan; ^4^Osaka City University Center for Health Science InnovationOsaka, Japan; ^5^Department of Neurosurgery, Osaka City University Graduate School of MedicineOsaka, Japan; ^6^Wellcome Laboratory of Neurobiology, University College LondonLondon, UK

**Keywords:** dopamine, orbitofrontal cortex, medial prefrontal cortex, positron emission tomography (PET), romantic love

## Abstract

Using [^11^C]raclopride, a dopamine D_2_/D_3_ receptor antagonist, we undertook a positron emission tomography (PET) study to investigate the involvement of the dopaminergic neurotransmitter system when subjects viewed the pictures of partners to whom they were romantically attached. Ten subjects viewed pictures of their romantic partners and, as a control, of friends of the same sex for whom they had neutral feelings during the PET study. We administered [^11^C]raclopride to subjects using a timing for injecting the antagonist which had been determined in previous studies to be optimal for detecting increases in the amount of dopamine released by stimulation. The results demonstrated statistically significant activation of the dopaminergic system in two regions, the medial orbitofrontal cortex (mOFC) and medial prefrontal cortex, the former of which has been strongly implicated in a variety of rewarding experiences, including that of beauty and love. A positive correlation was obtained in mOFC between excitement levels and dopaminergic activation only in the love but not in the control condition.

## Introduction

Love, especially during its passionate phase, is an all-encompassing experience which, despite its disorienting effects, is regarded as a highly pleasurable and rewarding one by most. The experience of love when viewing the face of a loved partner, whether of the opposite or the same sex, correlates with activity in a small number of distinct cortical and subcortical areas of the brain (Bartels and Zeki, 2000; Fisher et al., 2005; Zeki and Romaya, 2010), which is not to say that these areas act in isolation but only that they are especially prominently engaged during such experiences. This restriction in the number of areas prominently engaged is surprising, given the all-embracing nature of romantic love. Yet it is also an advantage for it limits the areas to be explored for other neural characteristics that correlate with the experience of love—at least in initial exploratory studies. The relatively small number of areas involved encouraged us to extend earlier brain imaging studies and enquire whether there is any particular neurotransmitter activity that is especially prominent in them under similar conditions. The obvious choice in this initial study was dopamine, partly because it has been found to be critical for pair-bonding in voles (rodents) (Young and Wang, 2004; Mcgraw and Young, 2010), even if it does not act in isolation but as part of a critical neurotransmitter circuit that also includes oxytocin and vasopressin, and partly because it has been closely associated with reward (Schultz, 2001; Jonasson et al., 2014).

Our aim was to learn whether there is any increase in dopamine release during phases of romantic love in humans, and whether the increase could be localized to specific areas of the brain. Although dopamine neurons are located in the ventral tegmental area (VTA), nucleus accumbens and other parts of the midbrain and brain stem, the dopaminergic system has rich connections with a number of subcortical and cortical terminals, including the orbitofrontal cortex (OFC) of the forebrain. Any of the many areas that are rich in dopaminergic activity could therefore be involved. The medial orbito-frontal (mOFC) cortex was especially interesting since it is active in conditions in which maternal love is expressed (Bartels and Zeki, 2004), when sexually attractive faces are viewed (Ishai, 2007) and, more generally, during rewarding experiences (Schultz, 2001). We were particularly interested in D_2_ receptors because their activation has been reported to accelerate rewarding experiences, at least in voles, one of the most intensively studied species in terms of social and pair-bonding (Young and Wang, 2004). Mating in them results in an increase in extracellular dopamine in the nucleus accumbens while administration of dopamine antagonists blocks mating preferences. This made it plausible to suppose that, likewise in humans, the rewarding experience of viewing pictures of loved partners will similarly result in increased dopaminergic D_2_ receptor activity.

## Materials and methods

### Subjects

We recruited 13 healthy volunteers who were in the early stages of romantic heterosexual relationships; all had normal or corrected-to-normal visual acuity, were right-handed according to the Edinburgh handedness inventory (Oldfield, 1971) and had no history of medical illness. They all gave written informed consent. Three male subjects were subsequently excluded from the analysis because two moved during the scanning experiments and there was a failure in the synthesis of the PET tracer in one. Thus, the total number of subjects was 10 (6 females and 4 males, with an average age of 27.4 ± 4.3 years). The durations of the partnerships were in the range of 2–125 months (median = 17 months).

During a first visit to the laboratory, some 2 weeks prior to scanning, each subject provided 8 picture portraits of their partner, taken from different angles, and a similar number of portraits of other friends of the same sex as their partner, whom they had known for equivalent periods but toward whom they had neutral feelings. All pictures were matched as far as possible for expression and general appearance. The experiment was explained to the subjects, who viewed a sample stimulus of a random anonymous face. Each subject completed a Passionate Love Scale (PLS) (Hatfield and Sprecher, 1986; Hanari and Kawano, 2012) questionnaire, to attempt to quantify their feelings about their partner as far as possible. Age and length of relationship were recorded for each subject. After the PET scan, subjects recorded their subjective sensation of excitation on a visual analog scale (VAS) from 0 (no excitation) to 100 (most excitation), in order to ascertain the intensity of their feeling immediately after the scans. The entire protocol was approved by the Ethics Committee of the Osaka City University Graduate School of Medicine.

### Experimental design and positron emission tomography (PET)

**Figure 1:**
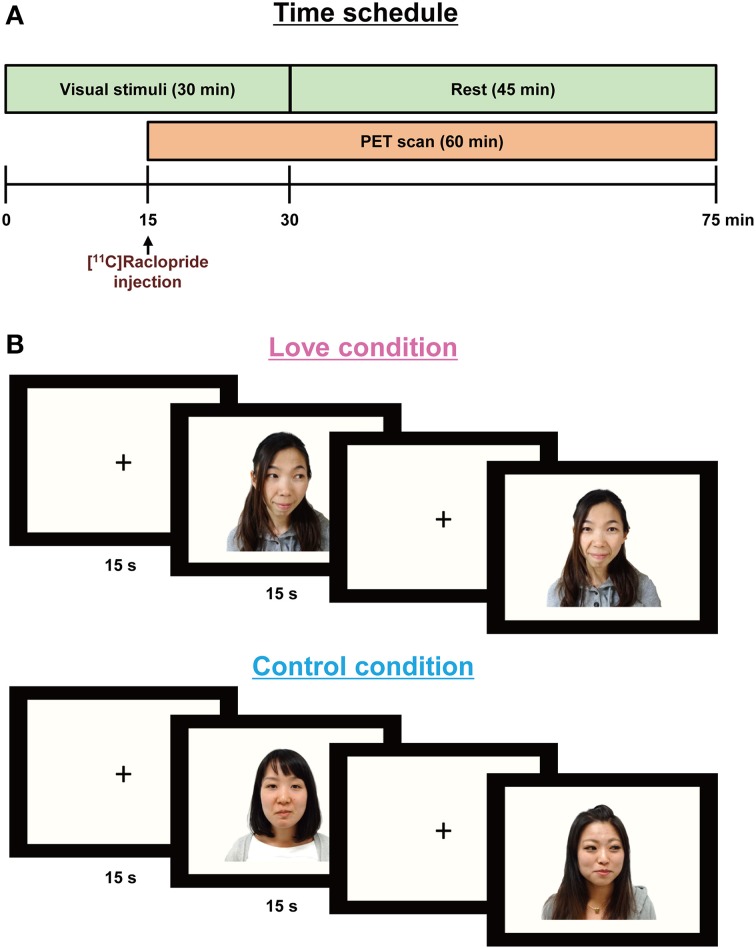
**Paradigm for the PET experiments (A) showing stimulus display sequences (B)**. In order to allow for release of endogenous dopamine, the visual stimuli were presented 15 min before [^11^C]raclopride administration and lasted for 30 min. A PET scan was conducted for 60 min after the [^11^C]raclopride administration. Each partner's or friend's picture was presented for 15 s, followed by a baseline blank epoch for 15 s.

The time schedule of experiments is shown in Figure 1A. Two PET scans were performed to measure the availability of dopamine D_2_/D_3_ receptors in the brain during the experience of love (i.e., while watching the partner's pictures) and during the control condition (while watching friends' pictures). Subjects viewed stimuli corresponding to one condition in the morning and to the other in the afternoon, the conditions being counterbalanced across subjects. Subjects were positioned in the PET scanner (Biograph-16, Siemens, resolution 4.6 × 4.6 × 5.1 mm) with their heads tilted slightly upwards and fixed to enable them to view comfortably the monitor used for presenting the stimuli; the heads were lightly tied with bandages to minimize movement during the scans.

Subjects were required to press a key each time a picture appeared on the monitor screen, to ensure consistent attention over the duration of the experiment. Before the emission scans, CT scans were performed for head positioning and attenuation correction. The PET scan procedure followed a previous study aimed at detecting dopamine turnover during the aesthetic experience of music (Salimpoor et al., 2011); briefly, in order to allow for release and accumulation of endogenous dopamine, the visual stimuli (pictures) were presented 15 min before the administration of the dopamine antagonist, [^11^C]raclopride, and lasted for 30 min.

*Stimuli*: Visual stimuli were created by clipping the outlines of the face and body in the original photograph images provided by each subject, and replacing background details with a flat mid-gray tone (Zeki and Romaya, 2010). In the love condition, subjects viewed their partners' picture, in random order, for 15 s; this was followed by a baseline blank epoch for 15 s (Figure 1B). For the control condition, they viewed the pictures of friends, presented again in random order. Each scanning session consisted of 60-pictures and 60-blank epochs, over a total period of 30 min. Subjects closed their eyes while remaining awake for 45 min after the presentation of the visual stimuli.

[^11^C]Raclopride was synthesized at Osaka City University Hospital by methylation using desmethyl raclopride, as described previously (Langer et al., 1999). The identity and concentration of [^11^C]raclopride were assessed using high-performance liquid chromatography. The purified fraction was evaporated and reconstituted with approximately 5 mL of saline containing 100 mg ascorbic acid. The radiochemical purity was more than 99%, and the specific radioactivity was 16.7 ± 9.7 GBq/μmol at the administration. The dose of administered [^11^C]raclopride was 5.4 ± 1.5 MBq/kg B.W. At the start of the emission scan, [^11^C]raclopride was intravenously administered for ca. 30 s, and the catheter line was flushed with 15–20 mL saline to prevent radiotracer retention. Serial PET scanning of the brain was performed for 60 min in the 3-dimentional dynamic mode, in the following frames: 6 × 10 s, 6 × 30 s, 11 × 60 s, and 15 × 180 s.

### Magnetic resonance (MR) image

Fine structural whole-brain T1-weighted magnetic resonance anatomical images were acquired at the end of the experimental session for each subject, using the Philips Achieva 3.0 TX (Royal Philips Electronics). (Echo time, 2.48 ms; repeat time, TR = 1900 ms; TE = 4.62 ms; flip angle of 15°; FOV = 256 mm; one slab with number of 176 slices per slab; voxel dimensions, 1.0 × 1.0 × 1.0 mm)

### Analysis of PET data

PMOD software Version 3.5 (PMOD Technologies Ltd.) was used for quantitative analysis of PET data. PET and structural MR images were co-registered. The binding potential (BP_ND_) was calculated using the simplified reference model 2 (Wu and Carson, 2002), with the cerebellum as the reference region. Using Statistical Parametric Mapping 8 software (SPM8, Wellcome Department of Imaging Neuroscience), whole-head structural images were then normalized to the Montréal Neurological Institute (MNI) T1 image template, with the same parameters applied to the BP_ND_ image. BP_ND_ images were resampled to a voxel size of 2.0 × 2.0 × 2.0 mm, using SPM8. The between-condition comparison (love vs. control) of the BP_ND_ was performed on a voxel-by-voxel basis using *t* statistics, with the statistical threshold set at *P* < 0.001 at the voxel level and more than 20 voxels at the cluster level for the entire brain. The co-ordinate of each brain region was defined by using the Wake Forest University Pick-atlas (Maldjian et al., 2003).

### Physiological responses

Objective measures of physiological responses indicative of excitation were collected during the experiments with the Procom Infinity biofeedback system by Thought Technology (Montreal, Canada). Participants' right hands were attached to the recording equipment to monitor heart and respiration rates, skin conductance and temperature, blood volume pulse (BVP) amplitude, and body temperature, as described previously (Salimpoor et al., 2011). In order to evaluate sympathetic and parasympathetic activity, low frequency (LF), and high-frequency (HF) components of the pulse-interval variation of the BVP (which reflect sympathetic and parasympathetic nerve activities, respectively, Malliani et al., 1991), were analyzed with fast Fourier transform. The LF power was calculated as the power within a frequency range of 0.04–0.15 Hz, and the HF one as that within a frequency range of 0.15–0.4 Hz. We analyzed averaged values for each physiological indicator for a 15 min period, starting from the beginning of the PET scan to the end of the stimulus presentation. Data for physiological responses were not obtained in one subject due to the failure of the measuring equipment.

### Statistical analysis

Differences between conditions for VAS parameters, physiological responses and BP_ND_ (plots) were analyzed using a paired *t*-test. Pearson's correlation analysis was also performed between these parameters. All *P*-values were two-tailed, and *P* values less than 0.05 were considered significant. These analyses were performed with the IBM SPSS 20.0 software package (IBM Corp., Armonk, NY).

## Results

### Subjective excitation and physiological response

We undertook scanning experiments, using PET to detect the activation of dopaminergic system, while 10 subjects, with an average score of 201.9 ± 33.7 on the PLS (Hatfield and Sprecher, 1986; Hanari and Kawano, 2012), viewed pictures of their romantic partners and, as a control condition, pictures of friends of the same gender as their partner but for whom they had neutral feelings (Figure 1). The results of the VAS, which is a measure of excitement levels when viewing pictures of romantic partners and friends, are summarized in Table 1, as are physiological responses that give objective measures of excitation. VAS value for excitation during the love condition was significantly higher (*P* < 0.001) than that of the control condition. In order to evaluate sympathetic and parasympathetic nerve activity during the two conditions, low frequency (LF) and high-frequency (HF) components of the pulse-interval variation of the blood volume pulse (which reflect sympathetic and parasympathetic nerve activities, respectively, Malliani et al., 1991), were determined. We analyzed averaged values for each physiological indicator for a 15 min period, starting from the beginning of the PET scan to the end of the stimulus presentation. Although the HF was similar between the two conditions, the LF component of autonomic activity during the viewing of romantic partners was significantly greater (*P* = 0.033) than that of the control condition, indicating that sympathetic nerve activity was enhanced by the viewing of partners' pictures. Other physiological indicators did not differ between the two conditions (Table 1).

**Table 1:** **Subjective excitation and physiological responses**.

	**Love**	**Control**	***p*-value**
VAS for excitation, score	55.3 ± 17.4	14.8 ± 9.6	<0.001
LF, ms^2^	60.9 ± 17.0	48.1 ± 21.0	0.033
HF, ms^2^	71.8 ± 24.4	62.8 ± 23.4	0.249
Skin temperature, °C	32.9 ± 2.6	34.2 ± 1.3	0.114
Skin conductance, μS	1.7 ± 1.0	1.8 ± 0.9	0.662
Heart rate, beats/min	61.7 ± 6.9	63.1 ± 8.2	0.254
Respiration rate, breath/min	7.0 ± 0.8	6.9 ± 0.8	0.699

*VAS, visual analog scale; LF, low frequency power; HF, High frequency power. Values are presented as mean ± SD*.

### Dopamine dynamics

**Figure 2:**
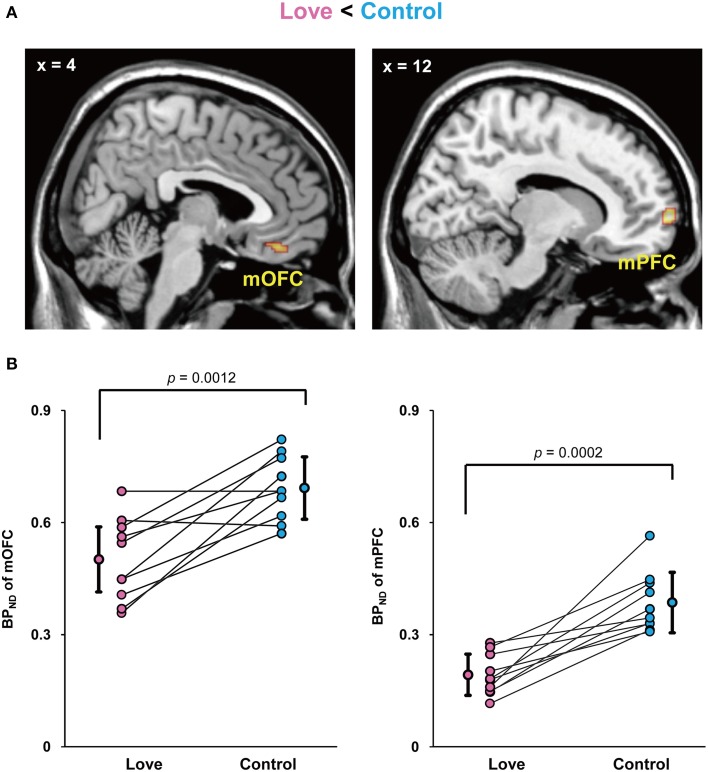
**Decreased BP_ND_ by viewing loved partners' pictures**. Statistical parametric maps of BP_ND_ in the medial orbitofrontal cortex (mOFC) and medial prefrontal cortex (mPFC) in the love condition (Control *minus* Love) **(A)** and graphs to show the peak value of the binding potential within the cluster (BP_ND_) in these regions of each subject **(B)**. The mean and standard deviation is shown in **(B)** and the *P* values resulted from statistical analysis by two-tailed paired *t*-test.

Since current technologies are not equipped to detect and measure dopamine release directly in humans, the usual solution is to employ an indirect measure of changes of dopamine release in the dopaminergic synaptic cleft during excitations produced during subjective state, in our case produced by viewing pictures of loved partners. We therefore injected subjects with a dopamine D_2_/D_3_ receptor antagonist, [^11^C]raclopride, with the aim of detecting detect increased amount of extracellular dopamine compared to baseline. We undertook a whole-brain search for differences in binding potential (BP_ND_)of raclopride, the index of dopamine D_2_ receptor binding, between the two conditions. The imaging results for the BP_ND_ between the love and control conditions (Control *minus* Love) are shown in Figure 2. The BP_ND_ of the (mOFC) and medial prefrontal cortex (mPFC) was significantly lower in the love condition than in the control condition, respectively (mOFC, *P* = 0.0012; mPFC, *P* = 0.0002), indicating that the activation of dopaminergic system (increase in endogenous dopamine release) occurred when viewing pictures of loved partners (Figure 2A).

**Figure 3:**
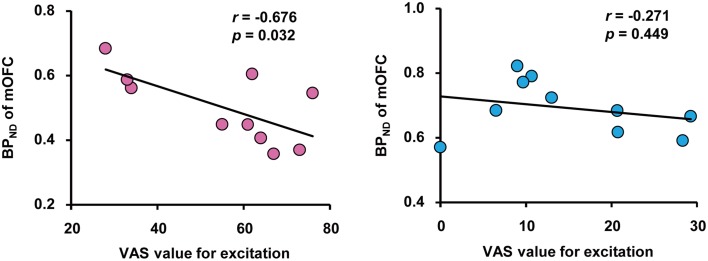
**Correlation between subjective excitement levels and BP**_ND_. Correlation between visual analog scale (VAS) values for excitation produced by viewing partners' (left) and friends' (right) pictures, and the BP_ND_ in the OFC during the love and control condition, respectively. Pearson's coefficient value and *P*value are shown. Note the absence of a correlation in the right panel.

We performed correlation analyses between BP_ND_ of brain regions related to passionate love, physiological parameters and VAS for excitation. This revealed that there was a significant negative correlation between VAS value and BP_ND_ in mOFC, but not in mPFC, in the love condition, indicating a correlation between intensity of subjective excitation and activation of dopaminergic system (Figure 3). There was no such correlation between VAS value and BP_ND_ in either cortical region during the control condition. There were no other significant correlations including inverse contrast (Supplemental Table 1).

## Discussion

We undertook this study to learn something about the neural correlates of romantic experiences at a molecular level. Our choice of the neurotransmitter to focus on, and brain areas to search for changes in, was facilitated by previous studies which have addressed the question of brain activity during attachment, pair-bonding and romantic love. A long line of studies in animals, and in particular the vole (Young and Wang, 2004; Mcgraw and Young, 2010), has established a highly specific turnover of neuro-hormones during pair bonding and social interactions, which encouraged us to suppose that we would, likewise, be able to detect specific neuro-hormonal activity that correlates with the experience of romantic love in humans. We opted to look at dopamine turnover, because dopaminergic activity in relation to mating and bonding has been relatively well studied. We also restricted ourselves to a study of the dopamine D_2_/D_3_ receptors within the dopamine family, because previous work in voles has shown that they are especially active in mate preference. We do not for a moment pretend that what we have presented here is anywhere like a complete picture of neurotransmitter activity during early phases of romantic love. Indeed, the close link between dopamine and other neurotransmitters such as serotonin makes of our study only a preliminary look at a vastly complex system.

We found a convincing difference in dopaminergic activity within the mOFC and mPFC between the control and love conditions. OFC is part of the emotional brain and has been implicated in a variety of emotional conditions, including the experience of beauty in general (Kawabata and Zeki, 2004; Ishizu and Zeki, 2011) beauty in a face (Ishai, 2007), maternal love (Bartels and Zeki, 2004), and aesthetic judgment (Ishizu and Zeki, 2013). It has also been repeatedly shown to be engaged during reward experiences (Gottfried et al., 2003). The part of the mOFC implicated in the present study corresponds closely to the part that was engaged during experiences of beauty and of maternal love, suggesting that the dopaminergic activity there may not be related uniquely to romantic love but may be more related to rewarding experiences, of which romantic love is one example. There could, nevertheless, be further subdivisions within the mOFC that are active during different types of reward. We were however interested to observe that, although both regions showed more dopaminergic activity, the correlation between VAS value and BP_ND_ was observed in the mOFC alone, suggesting a difference in the role of the two areas within the context of our experiment. The involvement of two distinct regions of the frontal cortex in our study, in one of which (mOFC) there was a (negative) correlation with VAS values while in the other there was none, may be indicative of a much wider functional sub-specialization within the OFC. Izuma et al. (2008), likewise found that the activity in mPFC was not graded in a self-evaluation paradigm, unlike activity in the striatum which correlated with reward level.

Thus, this first PET study of neurotransmitter activity during the experience of romantic love not only revealed specific areas of the brain where the dopaminergic system is activated during the experience, but additionally showed that different subdivisions within the frontal cortex may use this heightened release in different ways. Future studies should determine whether this specific pattern of dopaminergic activation in the mOFC is unique to romantic love or whether it is a more general pattern that correlates with other intense, and rewarding, emotional experiences. It would, as well, be interesting to determine the relationship between dopamine release in the OFC and dopaminergic activity elsewhere in the brain, particularly in areas of the subcortex that have been implicated in the experience of romantic love.

### Conflict of interest statement

The authors declare that the research was conducted in the absence of any commercial or financial relationships that could be construed as a potential conflict of interest.

## References

[B1] Bartels A. Zeki S. (2000). The neural basis of romantic love. Neuroreport 11, 3829–3834. 10.1097/00001756-200011270-00046 11117499

[B2] Bartels A. Zeki S. (2004). The neural correlates of maternal and romantic love. Neuroimage 21, 1155–1166. 10.1016/j.neuroimage.2003.11.003 15006682

[B2a] Fisher H. Aron A. Brown L. L. (2005). Romatic love: an fMRI study of a neural mechanism for mate choice. J. Comp. Neurol. 493, 58–62. 10.1002/cne.20772 16255001

[B3] Gottfried J. A. O'doherty J. Dolan R. J. (2003). Encoding predictive reward value in human amygdala and orbitofrontal cortex. Science 301, 1104–1107. 10.1126/science.1087919 12934011

[B4] Hanari T. Kawano K. (2012). The passionate love, positive, and negative feelings toward romantic interest: by use of Japanese Passionate Love Scale. J. School Cult. Infor. Stud. 12, 65–69 Available online at: http://ir.lib.sugiyama-u.ac.jp/dspace/handle/123456789/900

[B5] Hatfield E. Sprecher S. (1986). Measuring passionate love in intimate relationships. J. Adolesc. 9, 383–410. 10.1016/S0140-1971(86)80043-4 3805440

[B6] Ishai A. (2007). Sex, beauty and the orbitofrontal cortex. Int. J. Psychophysiol. 63, 181–185. 10.1016/j.ijpsycho.2006.03.010 16759727

[B7] Ishizu T. Zeki S. (2011). Toward a brain-based theory of beauty. PLoS ONE 6:e21852. 10.1371/journal.pone.0021852 21755004 PMC3130765

[B8] Ishizu T. Zeki S. (2013). The brain's specialized systems for aesthetic and perceptual judgment. Eur. J. Neurosci. 37, 1413–1420. 10.1111/ejn.12135 23373763 PMC3792471

[B9] Izuma K. Saito D. N. Sadato N. (2008). Processing of social and monetary rewards in the human striatum. Neuron 58, 284–294. 10.1016/j.neuron.2008.03.020 18439412

[B10] Jonasson L. S. Axelsson J. Riklund K. Braver T. S. Ogren M. Backman L.. (2014). Dopamine release in nucleus accumbens during rewarded task switching measured by [^11^C]raclopride. Neuroimage 99, 357–364. 10.1016/j.neuroimage.2014.05.047 24862078

[B11] Kawabata H. Zeki S. (2004). Neural correlates of beauty. J. Neurophysiol. 91, 1699–1705. 10.1152/jn.00696.2003 15010496

[B12] Langer O. Halldin C. Dolle F. Swahn C. G. Olsson H. Karlsson P.. (1999). Carbon-11 epidepride: a suitable radioligand for PET investigation of striatal and extrastriatal dopamine D2 receptors. Nucl. Med. Biol. 26, 509–518. 10.1016/S0969-8051(99)00005-0 10473189

[B13] Maldjian J. A. Laurienti P. J. Kraft R. A. Burdette J. H. (2003). An automated method for neuroanatomic and cytoarchitectonic atlas-based interrogation of fMRI data sets. Neuroimage 19, 1233–1239. 10.1016/S1053-8119(03)00169-1 12880848

[B14] Malliani A. Pagani M. Lombardi F. Cerutti S. (1991). Cardiovascular neural regulation explored in the frequency domain. Circulation 84, 482–492. 10.1161/01.CIR.84.2.482 1860193

[B15] Mcgraw L. A. Young L. J. (2010). The prairie vole: an emerging model organism for understanding the social brain. Trends Neurosci. 33, 103–109. 10.1016/j.tins.2009.11.006 20005580 PMC2822034

[B16] Oldfield R. C. (1971). The assessment and analysis of handedness: the Edinburgh inventory. Neuropsychologia 9, 97–113. 10.1016/0028-3932(71)90067-4 5146491

[B17] Salimpoor V. N. Benovoy M. Larcher K. Dagher A. Zatorre R. J. (2011). Anatomically distinct dopamine release during anticipation and experience of peak emotion to music. Nat. Neurosci. 14, 257–262. 10.1038/nn.2726 21217764

[B17a] Schultz W. (2001). Reward signaling by dopamine neurons. Neuroscientist 7, 293–302. 11488395 10.1177/107385840100700406

[B18] Wu Y. Carson R. E. (2002). Noise reduction in the simplified reference tissue model for neuroreceptor functional imaging. J. Cereb. Blood Flow Metab. 22, 1440–1452. 10.1097/00004647-200212000-00004 12468889

[B19] Young L. J. Wang Z. (2004). The neurobiology of pair bonding. Nat. Neurosci. 7, 1048–1054. 10.1038/nn1327 15452576

[B20] Zeki S. Romaya J. P. (2010). The brain reaction to viewing faces of opposite- and same-sex romantic partners. PLoS ONE 5:e15802. 10.1371/journal.pone.0015802 21209829 PMC3013131

