# Supplementary material for: Imaging the passionate stage of romantic love by dopamine dynamics
Source: Front Hum Neurosci. 2015 Apr 9;9:191. doi: 10.3389/fnhum.2015.00191 (PMC4391262; doi:10.3389/fnhum.2015.00191)
Supplement: Supplementary file 1 [file DataSheet1.DOCX]

1. **Supplemental Tupplemental dataemotional arousal, so did not specify to be sexual arousal.t excited as visual analog scale when they watched table 1.**

**Correlations among BP_ND_ of mOFC and mPFC in love condition, physiological parameters and VAS for excitation.**

|  | BP_ND_ of mOFC | |  | BP_ND_ of mPFC | |  | VAS for excitation | |
| --- | --- | --- | --- | --- | --- | --- | --- | --- |
|  | *r* | *P* value |  | *r* | *P* value |  | *r* | *P* value |
| VAS for excitation | -0.676 | 0.032 |  | -0.300 | 0.399 |  | - | - |
| LF | 0.068 | 0.862 |  | 0.135 | 0.728 |  | 0.072 | 0.855 |
| HF | -0.279 | 0.468 |  | 0.299 | 0.435 |  | 0.126 | 0.747 |
| Skin temperature | 0.061 | 0.876 |  | 0.618 | 0.076 |  | 0.315 | 0.409 |
| Skin conductance | 0.098 | 0.801 |  | 0.541 | 0.133 |  | -0.071 | 0.856 |
| Heart rate | 0.398 | 0.288 |  | 0.498 | 0.173 |  | -0.106 | 0.785 |
| Respiration rate | 0.531 | 0.141 |  | 0.432 | 0.245 |  | -0.028 | 0.942 |
